# The role of cell death in SARS-CoV-2 infection

**DOI:** 10.1038/s41392-023-01580-8

**Published:** 2023-09-20

**Authors:** Cui Yuan, Zhenling Ma, Jiufeng Xie, Wenqing Li, Lijuan Su, Guozhi Zhang, Jun Xu, Yaru Wu, Min Zhang, Wei Liu

**Affiliations:** 1 College of Life Sciences, Henan Agricultural University, Zhengzhou, China; 2 College of Pharmacy, Henan University of Chinese Medicine, Zhengzhou, China

**Keywords:** Infection, Microbiology

## Abstract

Severe acute respiratory syndrome coronavirus 2 (SARS-CoV-2), showing high infectiousness, resulted in an ongoing pandemic termed coronavirus disease 2019 (COVID-19). COVID-19 cases often experience acute respiratory distress syndrome, which has caused millions of deaths. Apart from triggering inflammatory and immune responses, many viral infections can cause programmed cell death in infected cells. Cell death mechanisms have a vital role in maintaining a suitable environment to achieve normal cell functionality. Nonetheless, these processes are dysregulated, potentially contributing to disease pathogenesis. Over the past decades, multiple cell death pathways are becoming better understood. Growing evidence suggests that the induction of cell death by the coronavirus may significantly contributes to viral infection and pathogenicity. However, the interaction of SARS-CoV-2 with cell death, together with its associated mechanisms, is yet to be elucidated. In this review, we summarize the existing evidence concerning the molecular modulation of cell death in SARS-CoV-2 infection as well as viral-host interactions, which may shed new light on antiviral therapy against SARS-CoV-2.

## Introduction

The pandemic of coronavirus disease 2019 (COVID-19) which is induced by severe acute respiratory syndrome coronavirus 2 (SARS-CoV-2), an emerging virus belonging to the coronavirus family, transitions the rarely studied viruses to the priority of global public health within several years. SARS-CoV-2, the single-stranded RNA virus of the β-coronavirus family, includes 29,903 nucleotides, which encode 16 non-structural proteins (NSP1–NSP16), nine putative accessory factors, along with four structural proteins: spike (S), envelope (E), membrane (M), and nucleocapsid (N). Cells and organs containing angiotensin-converting enzyme 2 (ACE2) surface receptors have been detected to be the major virus targets. Viruses can interact with ACE2 receptors and directly damage organs or worsen pre-existing systemic diseases.^1^

SARS-CoV-2 is related to the initial clinical manifestations mainly including non-specific respiratory syndromes, as well as complicated complications like acute respiratory distress syndrome (ARDS), acute lung injury (ALI), and multiple organ failure.^2–6^ There are two main signs of severe COVID-19, including the persistent interferon (IFN) response together with persistent viral RNA existence for several months.^7^ Consequently, several methods are put forward for combating COVID-19, like inhibiting inflammasome response, viral replication, as well as downstream IFN response.^8–10^ Overall, it is the focus of anti-COVID-19 intervention strategies to induce programmed cell death, through the immune system or relevant treatment.

With regard to multi-cellular organisms, a distinct immune defense mechanism is killing infected cells to protect the rest, which has an important effect on keeping homeostasis as well as disease pathogenic mechanisms. Main types of cell death, like apoptosis, necroptosis, proptosis, ferroptosis, and NETosis, have been recognized as the critical defense mechanisms against microbial infection. It has been reported that viruses usually stimulate diverse cell death types within diverse cells. Cell death has been increasingly suggested to be the double-edged sword of virus infection. Cell death at the medium level represents the protective intrinsic immune response, which could directly impede the spread of the virus by reducing replicative vehicles. In comparison, the out-of-control cell death will induce disordered immune responses, cytokine storm, tissue degradation, spreading of the virus, or even host death. SARS-CoV-induced cell death has been depicted in several tissues of infected cases, while different SARS-CoV components have been found to initiate cell death.^11–14^ Middle East respiratory syndrome coronavirus (MERS-CoV) induces cell death of different cell types and activates multiple cell death pathways.^14^ Recently, with the outbreak of novel COVID-19, researchers have found that SARS-CoV-2 causes cell death through various methods.

Cell death was once believed to be the result of a distinct and independent process. Nevertheless, a more intricate depiction of cell death modalities has emerged, elucidating the existence of crosstalk and backup mechanisms at the molecular level, thereby establishing connections among various pathways in recent years. For example, necroptosis acts as a supplementary system when apoptosis is hindered by infection, pharmacological agents, or genetic mutation.^15^ Moreover, the induction of glutathione peroxidase 4 (GPX4), a major regulator of ferroptosis, has been found to mitigate the occurrence of ferroptosis and pyroptosis during bacterial infection.^16^ Conversely, the degradation of glutathione (GSH) has been observed to augment necroptosis and ferroptosis in human triple-negative breast cancer cells, indicating a potential pathway that involves pyroptosis and necroptosis.^17^ Consequently, cell death pathways do not operate as separate entities acting in parallel. Instead, apoptosis, necrosis, pyroptosis, ferroptosis, and NETosis might be interconnected and cross-regulate each other.

The viral-host interaction should be further understood for the development of novel virus control methods. The present work focuses on summarizing the evidence regarding SARS-CoV-2-host cell interactions, especially for cell death. SARS-CoV-2-mediated cell death exerts a complicated effect on the antiviral immunity of the host, which may help with viral clearance or act as a mechanism for SARS-CoV-2-mediated tissue damage or disease development.^18^ Therefore, we propose the use of drugs to induce cell death as the novel candidate anti-SARS-CoV-2 agents. Such small molecules promote infected cell death with no influence on uninfected cells, consequently, limiting SARS-CoV-2 replication and transmission. Thus, it is a novel strategy to target virus-mediated cell death to treat SARS-CoV-2 infection.

## SARS-CoV-2 infection and apoptosis

### The machinery of apoptosis

Apoptosis means the programmed cell death form, which may occur due to cell damage or infection. Apoptosis mechanisms are classified as two major pathways, including intrinsic and extrinsic pathways. When extracellular ligands, including Fas ligand (FasL), trigger cell surface death receptors (like Fas) and then activate them, the death-inducing signal transduction complex (DISC) is formed, which contains Fas, Fas-associating protein with a novel death domain (FADD), together with procaspase-8, leading to caspase-8 activation. Thereafter, it activates caspase-3, eventually inducing an extrinsic apoptosis pathway. When DNA damage, cell stress, or intracellular signals are received, activation of the intrinsic apoptotic pathway can be achieved. Subsequently, apoptosis-promoting proteins from the pro-apoptotic B-cell lymphoma-2 (Bcl-2) family, Bcl-2 associated X (Bax), together with the Bcl-2 antagonist killer (Bak), induce changes in mitochondrial outer membrane permeability (MOMP), as a result, cytochrome c (Cytc) is released from mitochondria to the cytoplasm. In the cytosol, free Cytc forms an apoptotic complex called the apoptosome together with procaspase-9 and apoptotic protease activating factor-1 (Apaf-1), activating caspase-9 and leading to intrinsic apoptosis.

### SARS-CoV-2-encoded structural proteins induce apoptosis

Mounting evidence has demonstrated that apoptosis can be induced by structural proteins via several mechanisms. As a structural protein, M protein exhibits the highest abundance within SARS-CoV-2 particles, which has an important effect on maintaining virion size and shape.^19^ As suggested by studies using several cell lines, SARS-CoV-2 M may have a dual function in regulating apoptosis during infection. Yang et al. reported that the SARS-CoV-2 M protein showed direct inhibition on the ubiquitination of the Bcl-2 ovarian killer BOK (a pro-apoptotic protein), increased its stability without Bax and Bak, and then enhanced its mitochondrial translocation, thus inducing apoptosis of H292 cells through an intrinsic pathway.^20,21^ M protein overexpression-induced pulmonary injury is mitigated through BOK silencing.^22^ Additionally, the SARS-CoV-2 M protein hinders the pyruvate dehydrogenase kinase 1 (PDK1)-protein kinase B (PKB)/serine/threonine kinase (AKT) axis, which has an important effect against cell apoptosis through combining TM2 domain in M protein with PH domains in PDK1, thus reducing the substrate activities, which include apoptosis signal-regulating kinase (ASK) and forkhead transcription factor (FKHRL1).^23^ The reduced FKHRL1 phosphorylation level facilitates the nuclear translocation and promotes FasL level.

SARS‐CoV‐2 N protein, another viral protein with high abundance, is responsible for viral RNA replication and transcription, as well as ribonucleoprotein (RNP) complex formation and maintenance.^24–26^ Zhu et al. indicated that the M glycoprotein in SARS-CoV-2-induced caspase-mediated apoptosis based on N protein through interacting with PDK1 while hindering PDK1-PKB/AKT pathway activation.^27^ Besides, SARS-CoV-2 N protein is recently demonstrated the specific enhancement of M-mediated apoptosis by interaction with PDK1 and M, thereby further attenuating PDK1-PKB/AKT interaction in an M-dependent manner. As revealed by Ren et al.^23^ *N* interacted with PDK1 and M through diverse domains for forming the stable complex, while facilitating the M-mediated apoptosis. In addition, if specific peptides disrupt M-N interaction, it restores PDK1-PKB/AKT signaling and abolishes N’s role in enhancing M-mediated apoptosis.^23^ In addition, it was proposed that caspase-6 in the apoptotic cascade cleaves the N protein to produce an N fragment that acts as an IFN antagonist, thereby promoting viral replication.^28^

For SARS-CoV-2, its S protein contains S1 and S2 subunits, and both of them have an essential role in recognizing host cell receptor ACE2 and fusing cell and viral membranes, finally causing entry of viral cells.^29^ Jiang et al. described that the SARS-CoV-2 S protein was responsible for activating reactive oxygen species (ROS), which inhibit the phosphoinositide 3-kinase (PI3K)/AKT/mammalian target of rapamycin (mTOR) pathways,^30^ thereby inhibiting Bcl-2 protein and promoting the intrinsic apoptosis pathway.^31–33^ Additionally, infection with SARS-CoV-2 S protein increases the levels of caspase-3 and caspase-6 for inducing THP-1-like macrophage apoptosis, possibly regulated via the increased ROS as well as calcium production in cells.^34^ In recent studies, infection with SARS-CoV-2 is found to decrease pancreatic insulin content and production and promote β-cell apoptosis after SARS-CoV-2 S protein treatment or SARS-CoV-2 infection, which induces pancreatic dysfunction and leads to hyperglycemia or diabetes.^35^

### Apoptosis induction by SARS-CoV-2-encoded accessory proteins

ORF3a, a SARS-CoV-encoded accessory protein, is previously suggested to induce cell apoptosis.^36,37^ As shown in several latest studies, ORF3a in SARS-CoV and SARS-CoV-2 share close structures and functions. ORF3a is a viroporin in SARS-CoV-2; therefore, it forms the ion channel onto the cell membrane for regulating cell apoptosis and promoting the release of the virus,^38,39^ which also indicates that membrane association is needed for SARS-CoV-2’s pro-apoptotic effect.^20,21^ According to Kern et al.^40^, SARS-CoV-2 ORF3a adopted the new dimeric fold with a high conservation degree among Coronaviridae and formed non-selective calcium (Ca^2+^) permeable cation channels, which trigger erythrocyte apoptosis. Based on recent reports, SARS-CoV-2 ORF3a is localized in endoplasmic reticulum (ER) compartment, which can induce reticulophagy regulator 1 (RETREG1)-dependent reticulophagy via high mobility group box 1 (HMGB1)-beclin 1 (BECN1) pathway, leading to ER stress. Consequently, it can induce cell apoptosis, finally enhancing SARS-CoV-2 infection and replication, and promoting pro-inflammatory responses via ER functional reprogramming and reticulophagy.^41^ Further study revealed that SARS-CoV-2 ORF3a could directly activate caspase-8 through the extrinsic pathway, then cleave Bep intracellular delivery (Bid) to the truncated form of the activator protein Bid (tBid) by caspase-8, thereby inducing activation and release of mitochondrial Cytc along with the caspase-9.^42^ Similarly, SARS-CoV-2 ORF3b can enhance apoptosis through activation of caspase-8.^43^

As the type-I transmembrane protein that is 121 amino acids long, SARS-CoV-2 ORF7a contains one N-terminal domain, one Ig-like ectodomain, one hydrophobic transmembrane domain, and a typical ER retention motif.^44^ ORF7a has an important effect on protein trafficking in Golgi complex and ER.^45^ As suggested recently, SARS-CoV-2 ORF7a can recruit Bcl-XL (a survival-promoting factor) to ER via Lys117 and Lys119 (two C-terminal residues), thus promoting cell apoptosis.^46^ Therefore, ORF7a can activate the cellular ER stress response and enhance apoptosis. Conversely, ORF7a ubiquitination suppresses the Bcl-XL interaction and inhibits ORF7a localization in ER, thus restraining ER stress activation while rescuing cells.^46^ Recently, SARS-CoV-2 ORF7b, also co-localized within the ER, is related to immune responses in the host through up-regulating interferon-beta (IFN-β), interleukin 6 (IL-6) and tumor necrosis factor-alpha (TNF-α) expression via type-I IFNs regulatory factor 3 (IRF3) and signal transducer and activator of transcription 1 (STAT1) phosphorylation. This then up-regulates IFN-β, IL-6, and TNF-α levels, eventually accelerating TNF-mediated apoptosis of Vero E6 and HEK293T cells.^47^ Meanwhile, ORF6 can promote apoptosis by inhibiting STAT1 nuclear translocation.^48^

ORF9b in SARS-CoV-2 may modulate IFN together with apoptosis signaling through the interaction with translocase of the outer membrane 70 (TOM70).^49^ A previous study indicated that TOM70 is related to mitochondrial antiviral signaling (MAVS) protein activation, finally causing apoptosis in the case of viral infection.^50,51^ Recently, as discovered by Gordon et al.^49^, ORF9b targeted TOM70 to inhibit the MAVS signaling downstream. TOM70 is the mitochondrial import receptor necessary for MAVS to activate TBK1 and IRF3, together with the later RNA-sensing responses. It promotes Noxa (a pro-apoptotic BH3-only protein) and Puma transcription in a p53-independent manner, thereby activating the innate apoptotic pathway. Moreover, Bax and Bak are responsible for disrupting the mitochondrial membrane integrity, as a result, Cytc is produced, and caspase-9 apoptosome is formed.^52,53^

### Apoptosis inhibition via SARS-CoV-2

**Fig. 1:**
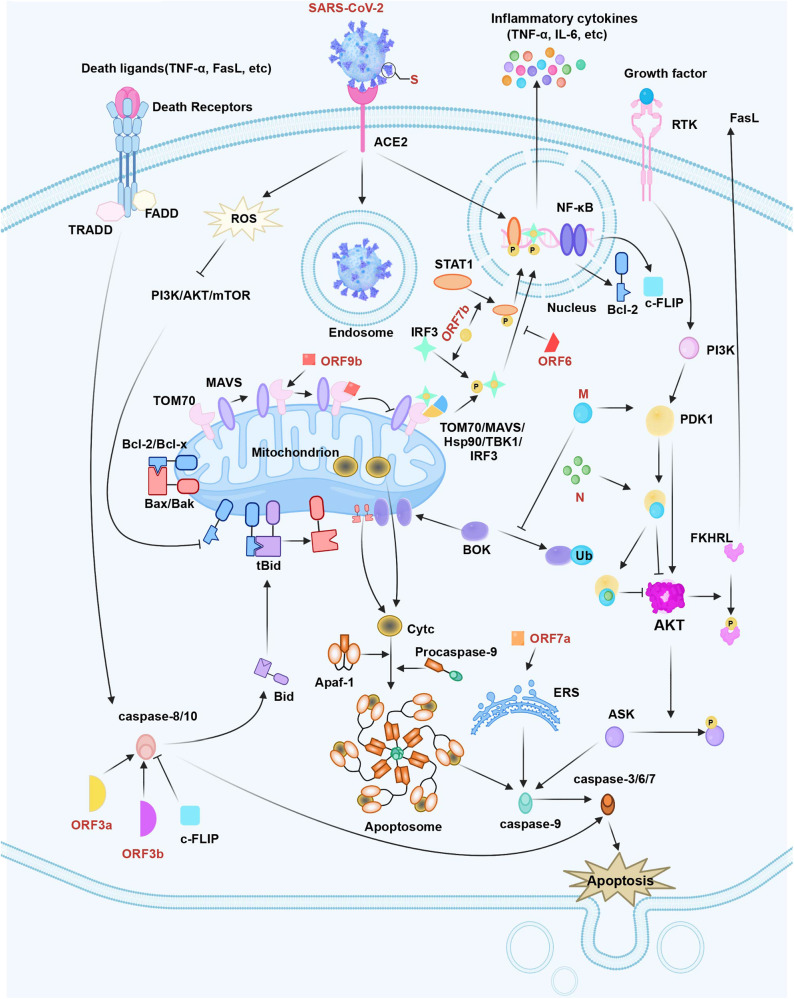
The apoptosis pathway in SARS-CoV-2 infection. SARS-CoV-2 S protein regulates Bcl-2 and Bax to trigger the intrinsic apoptotic pathway. SARS-CoV-2 M protein stabilizes BOK to trigger intrinsic apoptosis. Additionally, SARS-CoV-2 M interacts with PDK1 and inhibits the activation of PDK1-PKB/AKT signaling to induce caspase-dependent apoptosis. SARS-CoV-2 N improves M-induced apoptosis by interacting with M and PDK1, which can thus strengthen the M-mediated attenuation of PDK1-PKB/AKT interaction. SARS-CoV-2 ORF3a and ORF3b-activated caspase-8 can induce the apoptotic pathway. SARS-CoV-2 ORF6 hinders the nuclear translocation of STAT1. SARS-CoV-2 ORF7b promotes the phosphorylation and nuclear accumulation of IRF3 and STAT1, which activates TNF-α secretion and results in cellular apoptosis through the TNFR1 pathway. SARS-CoV-2 ORF7a recruits Bcl-XL to the ER, activating the cellular ER stress response and enhancing apoptosis. SARS-CoV-2 ORF9b suppresses signaling downstream of MAVS by targeting TOM70. In contrast, SARS-CoV-2 infection activates c-FLIP and NF-κB signaling to hinder apoptosis in infected cells

SARS-CoV-2 infection can raise FADD-like interleukin 1 (IL-1)-converting enzyme-inhibitory protein (c-FLIP) expression in cells, while the latter could regulate death effector domains for achieving caspase-8/10 deactivation.^54^ In a recent study, transcriptional output of the nuclear factor kappa B (NF-κB) pathway is found to be necessary for SARS-CoV-2 replication, therefore, SARS-CoV-2 infection is able to trigger the continuous and potent NF-κB transcriptional response.^55^ It is well known that the NF-κB pathway promotes the transcription of certain critical apoptosis inhibitors to obstruct apoptosis, like Bcl-2 and cellular c-FLIP.^56^ Proteomic analyses have shown that SARS-CoV-2 proteins, like ORF9c or NSP13, show interactions with NF-κB pathway.^57^ To be specific, it is discovered that SARS-CoV-2 S protein is responsible for activating the toll-like receptor (TLR)2-mediated NF-κB pathway within mouse and human lung epithelium.^58^ Therefore, SARS-CoV-2 may prevent clearance via apoptosis suppression, thus securing the sufficient time and location of early replication. Figure 1 displays the mechanisms of SARS-CoV-2 in the regulation of cell apoptosis.

### Apoptosis-targeted therapies in SARS-CoV-2 infection

SARS-CoV-2 has been suggested to potently promote different host cell apoptosis, leading to excessive tissue injury as well as loss of function, and this can facilitate disease occurrence and mortality in later stages. Therefore, blocking host cell apoptosis is necessary during early viral replication, and it has emerged as the candidate anti-COVID-19 therapeutic target.^22^ According to Feng et al.^59^, SARS-CoV-2 infection induced the excess immune response called the cytokine storm among severe COVID-19 cases. With regard to cytokine storm, inhibiting cell death via TNF- and IFN-γ-mediated pathways is the most direct management.^60^ Due to the engagement of TNF receptor 1 (TNFRl) by TNF, the signal molecule complex known as complex I is rapidly assembled, which is transient with tumor necrosis factor receptor type 1-associated death domain protein (TRADD) and is promptly released into the cytoplasm for forming the cytosolic TRADD/FADD/caspase-8 complex, called DISC.^61^

The apoptotic potential of DISC is restrained by c-FLIP, as it hetero-dimerizes with caspase-8 and blocks caspase-8 activation.^62^ Consequently, using small molecules targeting c-FLIP for activating caspase-8 while inducing extrinsic apoptosis is the critical way for contrasting viral replication.^63^ Infliximab, the human-mouse chimeric immunoglobin G1 (IgG1) monoclonal antibody (McAb) targeting TNF-α, has been applied in treating various autoimmune diseases. As found in a one-arm phase II trial, Infliximab administration reduced the pathological inflammatory cytokine levels among critical and severe COVID-19 inpatients (NCT04425538).^64,65^ Adalimumab, a recombinant fully-humanized anti-TNF-α McAb IgG, is used in combination with dexamethasone and remdesivir to treat severe COVID-19 cases. However, no positive results were found in ICU stay or mortality among severe COVID-19 cases.

In later stages, SARS-CoV-2 potently promotes different host cell apoptosis, leading to multiple tissue injuries together with loss of function. Therefore, blocking apoptosis pathways through suppressing the death receptor pathway as well as caspase cascade is the potential way for attenuating viral spread. Emricasan is a small molecule pan-caspase inhibitor that is administered orally, which can prevent caspase-3-dependent death of human cortical neural progenitors in the course of Zika virus infection.^66^ Plassmeyer et al.^67^ reported that emricasan suppressed the increased caspase-3 expression within samples cultured using plasma obtained from COVID-19 cases. Similarly, Q-VD, another pan-caspase inhibitor, strongly avoids caspase-3 activation. Importantly, according to André et al.^68^, Q-VD can prevent T-cell apoptosis while enhancing Th1 transcripts (IFN-γ, TNF-α) among COVID-19 cases.

During COVID-19 outbreak, research regarding whether Lianhua Qingwen (LHQW) was effective and safe in treating COVID-19 cases reported a markedly shorter recovery time, probably associated with the suppressed IL-6, IP-10, and TNF-α expression, whereas promoted inflammatory cell infiltration.^69,70^ Yang et al. revealed that LHQW attenuated lipopolysaccharide (LPS)-mediated ALI, with LHQW at a medium-dose exhibiting the best efficacy. Mechanistically, LHQW inhibited LPS-induced p53 up-regulation while suppressing p53-dependent innate apoptosis pathways through down-regulating Bax, caspase-3, and caspase-9, which elevated Bcl-2 levels but attenuated Cytc production among ALI mice.^71^ Similarly, lung cleansing and detoxifying decoction (LCDD) possesses effective components that alleviate inflammation and prevent cytokine storm initiation during COVID-19 via several pathways.^72^ Pirfenidone (PFD), a drug against fibrosis and inflammation, can suppress apoptosis, mitigate oxidative stress, decrease ACE receptor levels, reduce inflammation through multiple mechanisms, while simultaneously preventing COVID-19 invasion as well as the cytokine storm in pneumocytes.^73^

## SARS-CoV-2 infection and necroptosis

### Necroptosis machinery

Necroptosis represents the regulated cell death type under the mediation of mixed lineage kinase domain-like protein (MLKL) together with receptor-interacting serine/threonine kinase 1 or 3 (RIPK1/RIPK3). It can be triggered through the binding of ligands to virus sensors, pattern recognition receptors (PRRs), and TNF family death domain receptors. Binding with ligands, such as TNF-α or FasL, the death receptors further recruit TRADD, FADD, RIPK1, RIPK3, and caspase-8 for achieving necrosome assembly. In TNF-stimulated cells, inactive caspase-8 causes RIPK1-RIPK3 autophosphorylation, thereby phosphorylating and promoting the oligomerization of MLKL. pMLKL oligomerization forms pores in the cytomembrane and leads to necroptosis. Similar to necrosis, necroptosis features the morphology of necrosis, including cell swelling and rupture.^74^

### Necroptosis in SARS-CoV-2 infection

**Fig. 2:**
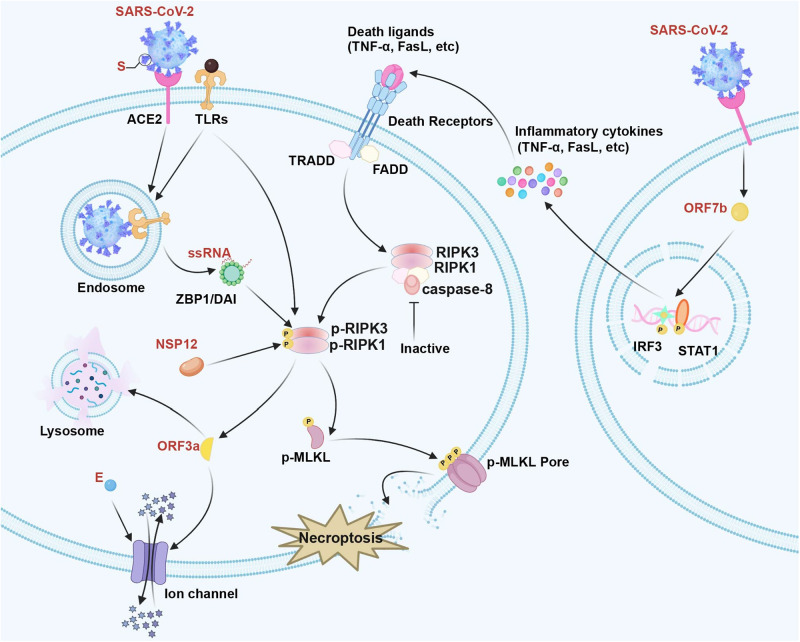
The necroptosis pathway during SARS-CoV-2 infection. SARS-CoV-2 ORF7b promotes the expression of death ligands (such as TNF-α, FasL, and IFN-γ). SARS-CoV-2 NSP12 can interact directly with and stimulate RIPK1 activation. SARS-CoV-2 ORF3a and E form a Ca^2+^ permeable cation channel to induce necroptosis. In addition, SARS-CoV-2 stimulates TLRs pathway and viral ZBP1 axis, directly activating RIPK3 to trigger necroptosis

Necroptosis is increasingly suggested to be related to COVID-19 pathogenic mechanism, based on the activation of RIPK1 observed within COVID-19.^1,75,76^ Xu et al. utilized lung pathological tissues from COVID-19 cases and cultivated SARS-CoV-2-infected human lung organoids for detecting RIPK1’s effect on SARS-CoV-2 infection.^77^ Additionally, they also discovered that SARS-CoV-2 NSP12 showed direct interaction with RIPK1 for facilitating the activation, thus inducing transcription of pro-inflammatory factors as well as host factors, such as ACE2, finally promoting virus to enter cells.^77^ Based on a previous study, SARS-CoV ORF3a shows selective K^+^ channel and membrane insertion activities, eventually causing cell death of different types (including necroptosis). Unlike SARS-CoV ORF3a, within the liposome system, SARS-CoV-2 ORF3a and E form the non-selective Ca^2+^ permeable cation channel, which promotes cell death via necroptosis and apoptosis.^78^ Moreover, SARS-CoV-2 immune complexes promote the necroptosis of monocytes RIPK3- and MLKL-dependently. Additionally, as discovered by Rothan et al., in SARS-CoV-2-infected mouse brains and neurons, ZBP1/pMLKL-mediated necroptosis pathway was activated.^79^ FNDC4 and FNDC5, the myokines of the fibronectin type III domain-containing family, reduce of SARS-CoV-2 entry points together with necrotic-like cell death resulting from spike glycoprotein S1 within human adipocytes.^80^ In a late research by Santos et al., SARS-CoV-2 immune complexes increased necroptosis of monocytes RIPK3- and MLKL-dependently. They also observed that blocking necroptosis-associated proteins (RIPK1, RIPK3, and MLKL) dramatically decreased SARS-CoV-2 N gene level within monocytes.^81^ Furthermore, Ferren et al. revealed that necroptosis occurred during SARS-CoV-2 infection within both lungs and the brainstem in hamsters.^82^ Necroptosis resulting from SARS-CoV-2 can be found in Fig. 2.

### Necroptosis-targeted treatments during SARS-CoV-2 infection

Necroptosis can block viral replication through promoting cell death, but on the contrary, it can promote viral spread as well as cellular content release via cell rupture. Therefore, it is a candidate anti-COVID-19 strategy to target the necroptotic pathway components. At present, RIPK1, RIPK3, and MLKL are extensively identified to be important targets specific to necroptosis mechanism. Proteome-wide data and RNA-sequencing data suggested that RIPK1 was the candidate anti-COVID-19 therapeutic targets for these cases. Primidone, which has been approved by FDA, can decrease TNFα-mediated inflammation in vivo and RIPK1-triggered necroptosis in vitro, and is thereby good for COVID-19 cases.^83^ In addition, Necrostatin-1 (Nec-1) accounts for the necroptosis inhibitor targeting RIPK1. Using Nec-1 and its analogs can mitigate the corresponding necroptosis signaling pathway and thus mitigate systemic inflammation, cytokine storm, as well as SARS-CoV-2 infection.^84,85^ Moreover, treatment with Necrostatin-1 (a RIPK1 inhibitor) decreased viral load, mitigated inflammation and injuries in the SARS-CoV-2-infected mouse and cell models.

## SARS-CoV-2 infection and pyroptosis

### Pyroptosis machinery

Pyroptosis accounts for the programmed cell death of inflammatory and lytic type, which shows the features of cell swelling, membrane pore formation, DNA condensation and fragmentation, the release of cellular contents, as well as cell lysis.^86,87^ It can be activated via inflammatory caspase, ultimately releasing pro-inflammatory factors. Inflammatory caspases, like caspase-1/4/5/11, can be recruited into inflammasomes that show higher molecular weights in the case of stimulation.^88^ As sensors for pathogens, inflammasomes can recognize PAMP and assemble different inflammasomes according to PAMP chemical properties. Consequently, caspase-1 can be activated within inflammasome, which further promotes pro-IL-1β and pro-IL-18 processing. Meanwhile, the activation of caspase-4/5/11 could cause gasdermin-D (GSDMD) cleavage.^89^ As for GSDMD protein, its N-terminal fragments can promote membrane pore formation, which disrupts the cell membrane, eventually leading to lysis.^90^

### Pyroptosis during SARS-CoV-2 infection

It can be speculated that inflammasome makes a major contribution to COVID-19 by providing excess inflammatory responses when the body is infected with SARS-CoV-2.^91^ The SARS-CoV-2-encoded proteins have been increasingly suggested to modulate inflammasome activity via diverse mechanisms. In previous studies, SARS‐CoV‐2 S protein induces NLRP3 inflammasome, which represents the multiprotein complex necessary for secreting pro‐inflammatory factor IL‐1β, and the latter has an important effect on the hyperinflammatory syndromes in COVID‐19, as well as selective activation and secretion of cytokines in macrophages obtained from COVID‐19 cases.^92^ As indicated by one work conducted on adipose tissues, the SARS-CoV-2 S protein subunit 1-treated adipocytes experience pyroptosis through activating the LRR, NACHT, along with PYD domain-containing protein 3 (NLRP3), which leads to apoptosis-associated speck-like protein containing a CARD (ASC) formation, caspase-1 activation, GSDMD cleavage, and interleukin-1beta (IL-1β) production.^80^ In addition, SARS-CoV-2 NSP6 can directly interact with ATPase proton pump component (ATP6AP1) to damage lysosomal acidification, causing stagnation of autophagic flux, thus contributing to activating NLRP3 inflammasome as well as pyroptosis of lung epithelial cells.^93^ According to a recent study, SARS-CoV-2 ORF3a is the bridge connecting NIMA-related kinase 7 (NEK7) with NLRP3, resulting in IL-1β production via the A549 lung epithelial cells.^94^ As indicated by Bertoni et al., ORF3a protein in SARS-CoV-2 activates NLRP3, which promotes ASC speck formation.^95^ Additionally, NSP1 and NSP13 of SARS-CoV-2 shows direct inhibition on active caspase-1 within macrophage-like THP-1 cells mediated by NLRP3 inflammasome.^96^

Furthermore, Pan et al. showed the direct interaction between SARS-CoV-2 N protein and NLRP3, which accelerated ASC recruitment and promoted the assembly of NLRP3 inflammasome, causing pro-inflammatory factor maturation and triggering pro-inflammatory responses within the cultured A549 and HEK293T cells.^97^ Moreover, the SARS-CoV-2 N protein can occupy GSDMD’s linker region within the infected human monocytes to prevent caspase-1 cleavage via GSDMD.^98^ Moreover, viral SARS-CoV-2 N protein is discovered to interact with I-kappa-b-kinase (IKK) and TGF-beta activated kinase 1 (TAK1) complexes to promote NF-κB pathway activation, which promotes the production of pro-inflammatory factors like procaspase-1,NLRP3, pro-IL-1β, and pro-IL-18, ultimately leading to pyroptosis.^99^ Like SARS-CoV-2 N protein, ORF7a and NSP6 showed interaction with TAK1, recruited NEMO, and induced NF-κB-dependent cytokine production.^99,100^ Nsp5 from SARS-CoV-2 can cleave porcine gasdermin-D (pGSDMD) at Q193-G194 junction for generating two fragments unable to trigger pyroptosis.^101^

**Fig. 3:**
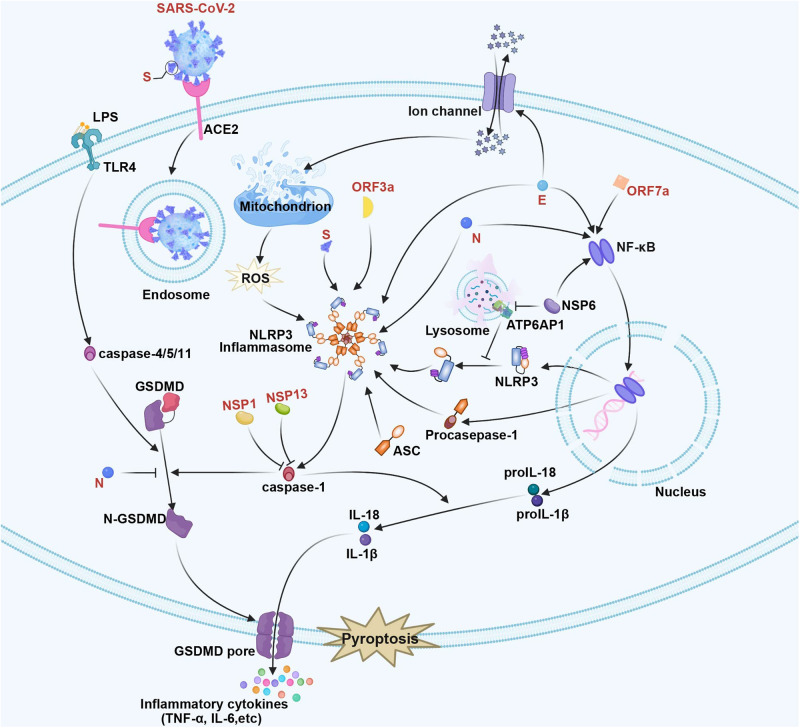
The pyroptosis pathway in SARS-CoV-2 infection. SARS‐CoV‐2 S triggers NLRP3 inflammasome activation to release the pro‐inflammatory cytokine IL‐1β. SARS-CoV-2 NSP6 stimulates the activation of the NLRP3 inflammasome by interacting with ATP6AP1, a vacuolar ATPase proton pump component. SARS-CoV-2 ORF3a and N facilitated NLRP3 inflammasome assembly induces ASC speck formation. In addition, SARS-CoV-2 N protein can protect GSDMD from caspase-1 cleavage. Moreover, SARS-CoV-2 N, NSP6, and ORF7a promote activation of the NF-κB pathway via interactions with TAK1 and IKK complexes, which stimulate pro-inflammatory cytokine production, containing pro-IL-1 β, pro-IL-18, procaspase-1, and NLRP3, which leads to pyroptosis. SARS-CoV-2 E can activate the NLRP3-dependent inflammasome and TLR2 pathways to trigger pro-inflammatory cytokines through activation of the NF-κB pathway. Additionally, SARS-CoV-2 E activates the NLRP3-dependent inflammasome and TLR2 pathways and forms a cation channel to trigger rapid cell death. SARS-CoV-2 NSP1 and NSP13 hinder the pyroptosis of infected cells by inhibiting the activity of caspase-1

As discovered by Zheng et al., SARS-CoV-2 E protein activated NLRP3-dependent inflammasome and TLR2 pathways, which induced pro-inflammatory factor generation by activating NF-κB pathway.^97,102^ Furthermore, SARS-CoV E glycoprotein was previously found to be protected from unfolded protein response stress-induced apoptosis, which promoted enhanced membrane reorganization, inhibited host translation, and optimized the environment to facilitate viral replication.^103^ Like the pyroptosis and necroptosis executors (GSDMs and p-MLKL separately), which form channels or pores to destroy membrane integrity, SARS-CoV-2 E protein is also found to generate the cation channel for inducing rapid death of multiple vulnerable cells, as well as perform the strong cytokine/chemokine production within macrophages leading to in vivo and in vitro ARDS-like injuries.^103^ Figure 3 summarizes pyroptosis in SARS-CoV-2 infection.

### Pyroptosis-targeted treatments during SARS-CoV-2 infection

Currently, pyroptosis dependent on inflammasome makes a vital impact on COVID-19 occurrence, which may be the candidate therapeutic target. Inhibiting fundamental components of cell pyroptosis, like cytokines, GSDMD, and NLRP3 inflammasome, could be a potential therapeutic strategy. According to Zeng et al., targeting NLRP3 inflammasome via MCC950 (a specific inhibitor of NLRP3) might be the possible management of COVID-19-associated immunopathology.^8^ Notably, treatment with MCC950 inhibited SARS-CoV-2 N protein-triggered lung damage as well as cytokine generation.^97^ One phase II clinical trial on dapansutrile (the specific NLRP3 inflammasome inhibitor) is being conducted among moderate COVID-19 cases who suffer from the early cytokine release syndrome (NCT04540120).^104^ GSDMD-dependent pores act as selective channels for cytokine release and the executor of cell pyroptosis, which implies the possibility of inhibiting GSDMD as a treatment. FDA-approved disulfiram can suppress pyroptosis through suppression of GSDMD pore formation as well as Cys191/Cys192 covalent modification within for blocking pore formation in cells and animal models, finally suppressing IL-1β generation and pyroptosis.^105^ Inhibiting pyroptosis enhancers, such as the ACE/Angiotensin II (Ang II) and serine/threonine kinase (ATR) axes, could be another strategy for COVID-19 treatment. Telmisartan, an angiotensin receptor blocker, suppresses the binding of Ang to corresponding receptors, thus reducing Ang-ATR signaling. According to one phase-IV randomized, SoC-controlled study, telmisartan decreased mechanical ventilation requirements while reducing death 15/30 days during hospitalization among COVID-19 inpatients (NCT04355936).^106^

Pyroptosis induces multiple inflammatory factors generation, among which IL-1β can amplify inflammasome activation through the positive-feedback loop. Anakinra is an antagonist of IL‑1 receptor, which inhibits IL‑1α and IL‑1β activities, and is approved by the FDA to be used to treat adult COVID-19 inpatients with positiveness of COVID-19 with pneumonia and requiring supplemental oxygen. Xiong et al. revealed that anakinra blocked NLRP3 inflammasome-dependent pyroptosis, finally decreasing the death of mice infected with SASR-CoV-2.^107^ Canakinumab, the human monoclonal antibody targeting IL‑1β, is the effective treatment option for mild or severe non-ICU adult COVID-19 inpatients. Canakinumab administration contributes to the rapidly and persistently improved oxygenation degree without serious side reactions.^108^ IL-6 has been increasingly suggested to be related to cytokine storm as well as COVID-19 pathogenic mechanism.^109^ Gordon et al.^110^ found that for critically ill COVID-19 patients receiving organ support at ICUs, tocilizumab (an IL-6 receptor antagonist) treatment could improve patient prognosis (NCT02735707).

## SARS-CoV-2 infection and ferroptosis

### Ferroptosis machinery

Ferroptosis is the programmed cell death type discovered newly, which is not the same as apoptosis or necrosis, and shows the features of production of oxidative stress, such as ROS, from iron-dependent lipid peroxide accumulation.^111^ Overload of ferric ion accounts for a major factor causing lipid ROS accumulation, therefore, this cell death type is also called iron death. For cells suffering from ferroptosis, they are associated with the typical morphology of mitochondrial condensation, cristae disappearance, and later outer mitochondrial membrane rupture. In canonical ferroptosis, GPX4 defense fails at first, which causes excess lipid peroxidation or even cell death. Intracellular iron is stored by ferritin and exported outside cells by ferroportin. When GPX4 is unable to efficiently eliminate ROS, phospholipid hydroperoxides (PLOOHs) will cause ferroptosis with iron accumulation.^112^ At the same time, iron overload results in the production of mitochondrial ROS. Polyunsaturated fatty acids (PUFAs) can be converted into PLOOHs, which is termed lipid peroxidation, and is regulated via lipoxygenases (LOXs), lysophosphatidylcholine acyltransferase 3 (LPCAT3), and acyl-CoA synthetase long-chain family member 4 (ACSL4), and leads to ferroptosis.^113^

### Ferroptosis in SARS-CoV-2 infection

Ferroptosis is increasingly suggested to be related to the COVID-19 pathogenic mechanism. Typically, the signature of ferroptosis was initially reported in one COVID-19 male case aged 48 years who had cardiogenic shock and decreased lymphocytes in the blood.^114^ Thus, ferroptosis is considered a potential factor causing cardiac arrhythmias among COVID-19 cases. The changes in serum markers for iron metabolism, such as elevated serum ferritin and reduced iron levels, demonstrate that iron overload is related to severe COVID-19.^115,116^ Additionally, SARS-CoV-2 infection is suggested to activate hepcidin pathway, thereby inhibiting Fe^2+^ export and leads to the progression of ferroptosis.^117^

During COVID-19, pro-inflammatory factor levels, including IL-1β, NF-κB, and TNF-α, within the brain increase in cytokine storm, as a result, AQP4 is up-regulated to aggravate brain edema, in addition, such pro-inflammatory factors are also related to ferroptosis machinery.^118^ SARS-COV-2 infection-induced inflammation can greatly promote IL-6 production. Previously, IL-6 is found to directly facilitate transferrin assimilation and ferritin expression.^119^ Meanwhile, IL-6 also promotes hepcidin production, which eventually leads to cellular iron accumulation. Yet several lines of evidence indicate that the higher serum hepcidin level is associated with COVID-19 severity.

**Fig. 4:**
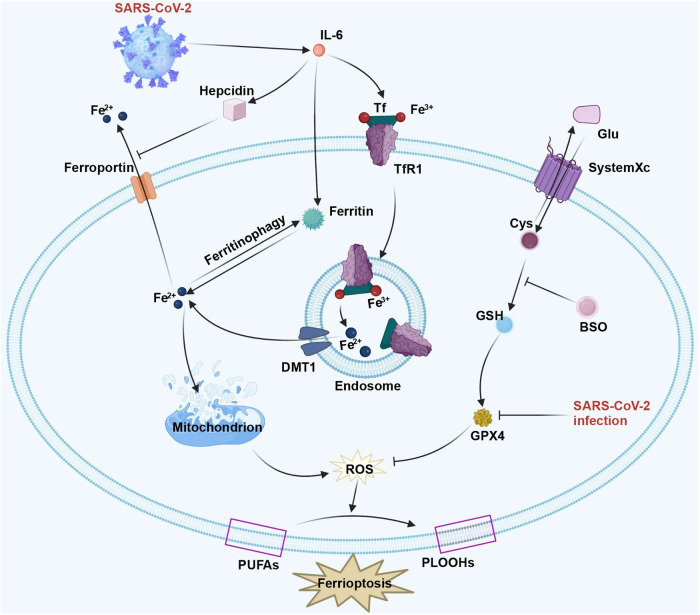
Ferroptosis pathway in SARS-CoV-2 infection. SARS-CoV-2 infection can activate the hepcidin pathway, inhibiting the output of Fe^2+^, resulting in ferroptosis. The expression of iron accumulation proteins transferrin and hepcidin is facilitated by the high level of IL-6 in COVID-19. Directly, this inhibits the exported protein ferroportin and causes cell ferroptosis. In addition, SARS-CoV-2 significantly reduced the expression of ferroptosis-related genes, containing GPX4, FTH1, FTL, and SAT1, which triggers lipid peroxidation and ferroptosis

Furthermore, in a previous study, African green monkey kidney (Vero) cells were infected with patient-derived SARS-CoV-2. According to their results, GPX4 mRNA expression markedly declined, which indicates that SARS-CoV-2 is associated with ferroptosis.^120^ Similarly, SARS-CoV-2 dramatically reduced GPX4 levels within brain cells, which raised the possibility that SARS-CoV-2 infection led to neurovascular events.^121^ As further found by Huang et al., expression of ferroptosis-associated genes (such as FTH1, GPX4, SAT1, FTL) within B cells, T cells, and PBMC in COVID-19 cases increased under disease conditions and decreased in the recovery phase.^122^ In addition, numerous investigations have verified that GSH is deficient in severe COVID-19 patients.^123,124^ Because of GPX4 deficiency, GSH is unable to undergo peroxidation for reducing lipid ROS produced by the Fenton reaction. Therefore, lipid peroxidation as well as ferroptosis can be triggered by lipid ROS accumulation. Further, severe COVID-19 cases with no pre-existing renal diseases may suffer from renal injuries. Thus, ferroptosis may be associated with renal symptoms in COVID-19.^125,126^ Figure 4 displays such complicated associations of SARS-CoV-2 with cell ferroptosis.

### Ferroptosis-targeted therapies in SARS-CoV-2 infection

It is the new research direction to investigate ferroptosis’ possible effect on COVID-19, which probably facilitates to development more therapeutic targets. As primary targets, the regulation of the iron load, GPX4-GSH axis, and lipid peroxidation may protect cells from ferroptosis. The FDA-approved iron chelator, imatinib can led to inactivation of iron-containing enzymes, inhibition of Fenton reactions, and blocking of SARS-CoV-2 infection-associated ferroptosis through binding to free iron^127^. Additionally, deferoxamine reduces IL-6 expression, which is an important inflammatory factor produced in COVID-19, therefore, it may be the candidate therapeutic against COVID-19-mediated liver injury. Nonetheless, other studies suggest the unfavorable effect of deferoxamine on COVID-19 patients, because iron chelators can deteriorate inflammation-induced anemia and weaken host inherent immunity.^128^ Additionally, more clinical trials of Desferal and tocilizumab as anti-COVID-19 iron chelation treatment are still underway (NCT04333550, NCT04361032, NCT04389801).^129^

With regard to the GPX4-GSH-cysteine axis, selenium (Se) is found to protect GPX4 against irreversible inactivation because of the effect on selenocysteine biosynthesis. As indicated by multiple lines of evidence, Se content shows positive relation to complications and clinical outcomes, supporting that Se should be supplemented among COVID-19 cases.^130–132^ In recent studies using the stroke mouse model, Se can promote GPX4 level while suppressing ferroptosis through strengthening protective transcriptional responses, and may be used in COVID-19 (NCT04869579, NCT04798677, NCT04751669, NCT04323228).^133^ Ebselen, the synthesized Se compound that mainly acts as a peroxiredoxin and GPX mimic, prevents ferroptosis as the GPX mimic and inhibits viral replication via the interaction with SARS-CoV-2 M protein.^134,135^

The cysteine prodrug, N-acetylcysteine (NAC) is utilized in the therapy for acetaminophen toxicity. In recent research, NAC is adopted to prevent COVID-19 or as an adjuvant treatment among COVID-19 cases. NAC is widely suggested to suppress ferroptosis. NAC partially rescued IL-6-promoted ROS production and ferroptosis of bronchial epithelial cells as well as nanoparticle-mediated ferroptosis of neurons, which partially interprets its roles in treating COVID-19.^136,137^ Therefore, NAC may be the GSH precursor that can suppress ferroptosis through reinforcing GPX4-GSH-cysteine axis, but not preventing radical propagation directly as the radical scavenging agent. Based on available clinical and experimental analyses, NAC makes a role in various possible therapeutic target pathways, and is correlated with SARS-CoV-2 infection pathophysiology, nonetheless, further clinical studies regarding NAC in treating COVID-19 as a monotherapy or combined treatment are underway (NCT04455243, NCT04928495, NCT05074121, NCT04792021, NCT04374461, NCT04703036).

As lipid peroxidation is increasingly identified with an important function in ferroptosis, and ferroptosis is found to contribute to degenerative disorders together with COVID-19, a strategy aiming at lipid peroxidation inhibition has become the possible anti-COVID-19 approach. In addition, liproxstatin-1 (Lip-1) and ferrostatin-1 (Fer-1) are considered to be effective ferroptosis inhibitors due to their potent radical trapping ability and their ability to decrease PLOOH accumulation within PUFAs.^138^ Recently, Lip-1 and Fer-1 are rarely studied, however, more research is required to validate their roles in inhibiting ferroptosis within various disorders like COVID-19.^139^

## SARS-CoV-2 infection and netosis

### NETosis machinery

NETosis represents the neutrophil extracellular traps (NETs)-dependent regulated cell death form. In NETs, polymorphonuclear (PMN) granulocytes are responsible for producing net-like architectures of decondensed chromatin and proteases. There are two different NETosis types characterized so far, namely, NADPH oxidase 2 (NOX2)- and NOX-independent NETosis.^140,141^ Generally speaking, this process begins with the chemokine- or PRR-mediated activation of neutrophils, then ROS generation along with calcium redeployment, which then activates protein arginine deiminase 4 (PAD4), the intracellular enzyme related to arginine residue deamination in histones.^142^

### NETosis within SARS-CoV-2 infection

NETosis primarily occurs upon bacterial or fungal infections; however, parts of viruses, like human immunodeficiency virus (HIV), Pox virus, and Hantavirus, initiate NETosis as well, which exhibits antiviral characteristics.^143–145^ Increasing studies have indicated significant increases in neutrophil counts, and they are related to SARS-CoV-2 infection severity. Notably, the pathogenic mechanism of COVID-19 is closely associated with body’s hyperinflammatory response, which is associated with pathological cytokine contents. It is well known that pro-inflammatory factors (IL-1β, IL-6, IL-8) can effectively recruit and activate neutrophils.^146^ Mounting evidence has suggested that pro-inflammatory factors like IL-1β or IL-8, represent the key mediators to induce NET, and they are abundant in macrophages and epithelial cells infected with SARS-CoV-2, which increases intravascular neutrophil and tissue NETosis.^147,148^ Additionally, the IL-8 autocrine loop can be detected within peripheral and pulmonary blood neutrophils, and it can enhance NET generation while indicating COVID-19 severity.^149,150^

**Fig. 5:**
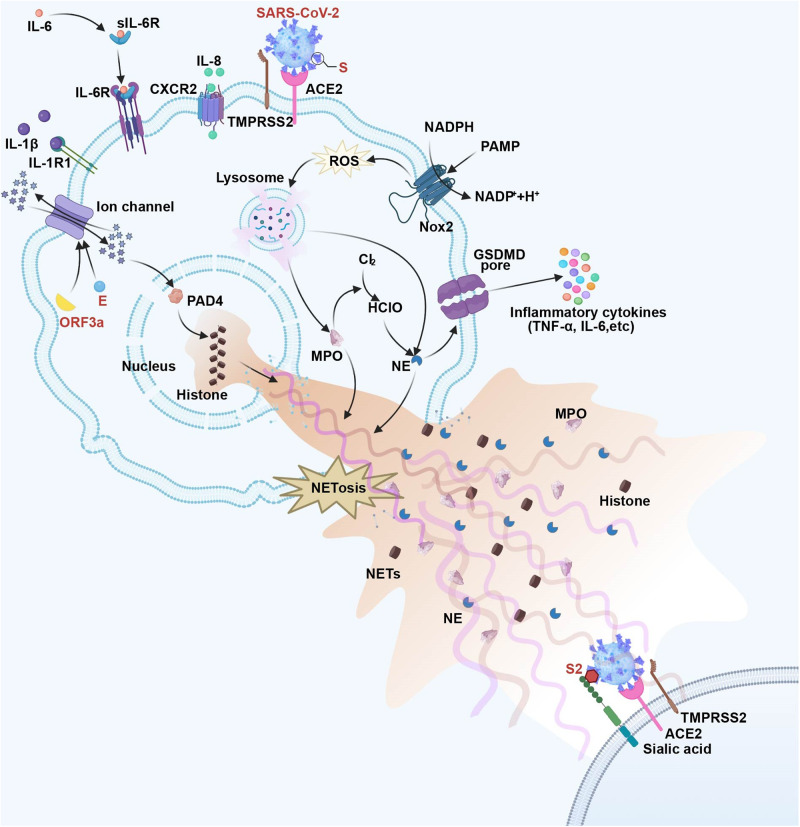
The NETosis pathway in SARS-CoV-2 infection. NETs, released by SARS-CoV-2-triggered NETosis, consist of NE, MPO, histones, and DNA. The IL-1β-IL-1R1, IL-6-IL-6R, and IL-8-IL-CSCR2 axes activate neutrophils. SARS-CoV-2 manipulates histones released to associate with S2 and sialic acid on the cell surface and promotes membrane fusion, ultimately enhancing its infectivity. Meanwhile, SARS-CoV-2 induces NETosis via increased ROS production, which is dependent on human ACE2, transmembrane protease serine 2 (TMPRSS2), and PAD4

Elevated blood neutrophil and NETosis levels can be detected among COVID-19 cases, besides, a high neutrophil-to-lymphocyte ratio has also been identified as the risk factor related to severe COVID-19.^151^ NETs contents within the plasma, lung tissue, and tracheal aspirate samples collected in COVID-19 cases increase relative to those in normal subjects.^152^ According to Arcanjo et al.^153^, SARS-CoV-2 stimulated NETosis of human neutrophils via promoting ROS generation. SARS-CoV-2-induced neutrophil infection may facilitate the direct activation of NETs generation under certain circumstances, which is dependent on human ACE2, PAD4, and transmembrane protease serine 2 (TMPRSS2). Reduced ACE2 levels increase Ang II concentration to decrease the protection, and this thus triggers pro-inflammatory milieu and oxidative stress under the action of NADPH oxidase.^154^ Consequently, NETs accumulation induces lung epithelial cell death.^155^ Recently, SARS-CoV-2 is found to regulate NETosis-produced histones (H3, H4), thus bridging S protein subunit 2 and cell surface sialic acid while promoting membrane fusion, ultimately enhancing its infectivity.^156^ Collectively, SARS-CoV-2-mediated NETosis can be a double-edged sword. It not only traps viral particles for effectively inhibiting transmission but also leads to cell damage and causes a cytokine storm.^157^ Figure 5 displays mechanisms of SARS-CoV-2 in regulating NETosis.

### NETosis-targeted therapies in SARS-CoV-2 infection

Targeting neutrophils together with NETosis is the possible applicable approach against COVID-19. For NETosis in COVID-19, three aspects for treatment have been considered: identifying key molecules involved in NETosis, preventing NET generation and decomposition, and blocking NETs expansion. Notably, treatments primarily concentrate on key factors related to NETosis, like neutrophil elastase (NE), PAD4, or GSDMD. Sivelestat, a selective NE inhibitor, is approved by the Republic of Korea and Japan to be used to treat acute lung injury. Based on one recent article, sivelestat achieved positive results on ARDSI cases, therefore, NE inhibitors are good for COVID-19 cases.^158^

PAD4, also a pivotal pro-NET enzyme, shows enhanced expression within COVID-19, and it is related to NET release^159^. Consequently, using PAD4 inhibitors, like Cl-amidine may impede both the SARS-CoV-2’s pro-inflammatory activities through attenuating NET generation.^160^ For instance, Cl-amidine can impair NETosis by reducing histone citrullination within blood neutrophils collected in COVID-19 cases and normal neutrophils infected with SARS-CoV-2.^152 Mo^reover, Cl-amidine can hinder lung epithelial cell apoptosis because of NET generation, indicating the feasibility of using PAD4 inhibitors for preventing lung injury as well as immunothrombosis during COVID-19.^152,160^ Besides, phase-III clinical studies on the PDE4 selective-inhibitors ensifentrine and apremilast on treating COVID-19 are underway (NCT04590586, NCT02735707).^161^

NETs degradation and elimination is also the appropriate pathway for investigation, thus reducing the NETs burden. As enzymes that degrade DNA, deoxyribonucleases (DNases) play critical roles in degrading NETs products. According to the latest clinical research during the treatment of COVID-19, treatment included DNase-I inhalation for dissolving thrombogenic NETs together with agents used to suppress cytokine-induced hyperinflammation, which achieved decreased in-hospital mortality, shortened length of hospital stay, decreased intubation rate, and even extended the overall survival (NCT05279391).^162^

As discovered by Egeblad et al., disulfiram administration decreased NET generation, perivascular fibrosis, and pulmonary inflammation within the golden hamster model of SARS-CoV-2 infection, which was achieved through decreasing the intrinsic immune and complement/coagulation pathways.^163,164^ Such antiproteases are undergoing preclinical or clinical trials for demonstrating their therapeutic effect on COVID-19.^165–167^ In terms of additional pathogens, SARS-CoV-2 can trigger the production of extracellular vesicles via platelets and activate C-type lectin receptor to promote NET generation.^168^ Consequently, suppressing C-type lectins is the potential treatment for reducing intravascular coagulopathy as well as the SARS-CoV-2-mediated NETosis.^169^ Mechanisms related to NET generation during COVID-19 should be further explored to develop more new treatments.^170^

Phosphodiesterases (PDEs) are enzymes related to the homeostasis of cyclic adenosine monophosphate (cAMP) as well as cyclic guanosine monophosphate (cGMP). PDE specific to cAMP shows high expression in neutrophils, which enhances inflammation. Therefore, some PDE inhibitors have been recognized to be potential therapeutics against COVID-19. Pentoxifylline has been approved by FDA to be a non-specific PDE inhibitor. As revealed by Liu et al., dipyridamole hinders the replication of SARS-CoV-2 within infected cells, alleviates disease severity, and markedly improves the prognosis of COVID-19 cases.^171^ Lymphocyte counts and serum lactate dehydrogenase (LDH) level can be easily obtained, which are related to COVID-19 severity, mortality, and need of hospitalization, and reflect the contribution of the host’s immunity to SARS-CoV-2 infection severity. However, higher lymphocyte counts together with lower serum LDH levels were detected among COVID-19 cases who were treated with pentoxifylline.^172^ One clinical study (NCT04433988) is ongoing to assess whether pentoxifylline is effective in treating COVID-19. Additionally, one phase Ib/II, randomized, placebo-controlled clinical trial on the efficacy of alvelestat (the new, oral small molecule for inhibiting neutrophil elastase) in treating COVID-19 complicated by ARDS has undergone phase I testing (NCT04539795).^173^

## Regulation of cell death in SARS-CoV-2 infection

Cell death resulting from viral invasion of host cells is a prevalent consequence following infection. It has traditionally been believed that cell death pathways function in parallel with little or no overlap. However, it is currently evident that cell death pathways are interconnected and capable of cross-regulation. The pivotal role of caspase as a mediator of apoptosis, necrosis, and pyroptosis was among the earliest identified bridges between various types of cell death. Caspase-8, in addition to its regulation of apoptosis, serves as a critical component in necroptosis. Most recently, accumulative studies have indicated that the activation of caspase-8 by SARS-CoV-2 ORF3a and ORF3b can trigger the apoptotic pathway. However, the role of caspase-8 in mediating SARS-CoV-2-induced necroptosis has not been reported before.

According to Ma et al.^174^, the syncytia resulting from the fusion of cells expressing SARS-CoV-2 S protein and ACE2 undergo pyroptosis, accompanied by increased caspase-3/7/9 activity and GSDME cleavage. It is noteworthy that the absence of caspase-9 not only inhibits GSDME cleavage and cell death, but also eliminates the S2' fragment of SARS-CoV-2 S-Flag induced by cell-cell fusion, suggesting a connection between caspase-9 and SARS-CoV-2 S protein cleavage. Therefore, targeting caspase-9 may offer a promising approach to preventing syncytia cell death. Consistent with this notion, the administration of the caspase-9 selective inhibitor, z-LEHD-fmk, significantly reduced SARS-CoV-2-induced lung damage in the K18-hACE2 transgenic mouse model. This reduction was evident through decreased hemorrhage and infiltration of inflammatory cells, as well as a mitigated pro-inflammatory response within the lung.^175^ Additionally, Chu et al. determined that this effect was attributed to the intrinsic inhibition of apoptosis by z-LEHD-fmk. However, further investigation is required to determine whether apoptosis switches to pyroptosis.

Disulfiram, an FDA-approved drug for alcoholism prevention, has been suggested as a potential therapeutic target for SARS-CoV-2 infection in Phase II trials by specifically targeting the SARS-CoV-2 main protease, 3CLpro.^176^ However, it has been discovered that disulfiram covalently binds to the human/mouse Cys191/Cys192 residues of the GSDMD protein, resulting in the inhibition of GSDMD pore formation, IL-1β release, and pyroptosis.^105^ Additionally, another study has demonstrated that disulfiram can impede NET formation and provide protection against SARS-CoV-2 infection in rodents.^163^ These observations collectively suggest that a single drug or compound may target multiple proteins across various signaling pathways, potentially leading to synergistic effects or unintended toxicities. Consequently, it is imperative to deeply investigate the types of cell death induced by SARS-CoV-2 infection, elucidate the underlying molecular mechanisms, and employ targeted pharmacological interventions to effectively mitigate the occurrence and prognosis of COVID-19.

## Conclusions and future perspectives

**Fig. 6:**
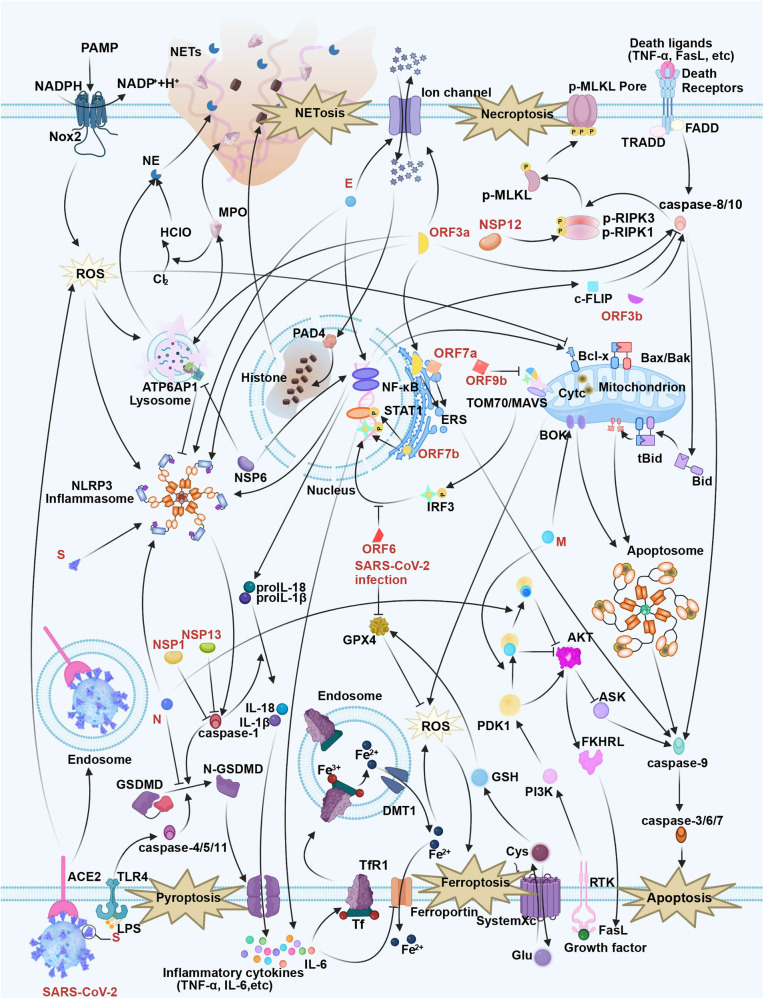
The sophisticated associations of SARS-CoV-2 and cell death: apoptosis, triggered by the extrinsic pathway (death receptor pathway) or the intrinsic pathway (mitochondrial pathway); necroptosis, mediated by RIPK1/RIPK3/MLKL; pyroptosis, induced by NLRP3 inflammasome, consisting of NLRP3, ASC, and caspase-1; ferroptosis, triggered by iron accumulation and overload, or ROS; NETosis, triggered by neutrophils and formed NETs to release of chromatin structures to neutralize intruders

The relationship between viruses and host cells involves complex interaction, while many cell and viral responses are related to viral infection as well as the associated pathogenic mechanism. Among them, host cell death has been the inherent immune defense mechanism against virus infections. Although cell death is an effective host defense strategy, hyperactivation of the antiviral response and inflammatory cell death can cause systemic inflammation and pathology. Therefore, the host must carefully balance cell death activation to prevent excessive inflammation while clearing the infection and blocking viral disease potentiation. Increasing evidence has indicated that SARS-CoV-2 modulates host cell death via multiple mechanisms (Fig. 6 and Table 1), which is also related to COVID-19 genesis and progression. Based on the above discussion, it can be concluded that cell death is a double-edged sword. For one thing, viral replication and transmission will be prevented since cell death can clear away infected cells. For another, uncontrolled cell death leads to uncontrolled cell injury along with an incompetent immune response, or even host death. Thus, careful consideration is required to determine the necessity and timing of inhibiting or activating cell death.

**Table 1:** The function of SARS-CoV-2 proteins in SARS-CoV-2 infection and cell death

Protein	Primary function	Type of cell death	Effect	Mechanism
M	Defines the shape of the virus envelope and maintains virion size and shape.	Apoptosis	(+)	Stabilizes BOK to induce intrinsic apoptosis.^20,21^
				Inhibits the PDK1-PKB/AKT axis to trigger caspase-dependent apoptosis.^23^
N	Packages the virus genome into RNP particles and inhibits the defense mechanisms of host cells.	Apoptosis	(+)	Enhances the M-induced apoptosis by interacting with M and PDK1.^23^
		Pyroptosis	(+)	Interacts with NLRP3, promotes ASC recruitment, and facilitates NLRP3 inflammasome assembly.^99^
			(+)	Promotes NF-κB activation by interacting with TAK1 and IKK complexes.^101^
			(−)	Protects GSDMD from caspase-1 cleavage.^98^
S	Binds to the receptor on the host surface and promotes cell invasion by the virus.	Apoptosis	(+)	Regulates Bcl-2 and Bax to induce the intrinsic apoptotic pathway.^31–33^
		Pyroptosis	(+)	Activates the NLRP3 inflammasome.^80,92^
		NETosis	(+)	Binds with histones released by NETosis and promotes membrane fusion.^156^
E	Promotes the packaging and reproduction of the virus.	Necroptosis	(+)	Forms a non-selective Ca^2+^ permeable cation channel.^78^
		Pyroptosis	(+)	Activates the NLRP3-dependent inflammasome and TLR2 pathways^97,102^ and forms a cation channel.^103^
ORF3a	Involves in SARS-CoV-2 virus release and the life cycle.	Apoptosis	(+)	Activates caspase-8 induces the apoptotic pathway.^42^
		Necroptosis	(+)	Forms a non-selective Ca^2+^ permeable cation channel.^78^
		Pyroptosis	(+)	Activates NLRP3 and inducing ASC speck formation.^94,95^
ORF3b	Blocks IRF3 nuclear translocation.	Apoptosis	(+)	Activates caspase-8 induces the apoptotic pathway.^43^
ORF6	Inhibits IFN production and IFN signaling.	Apoptosis	(+)	Inhibits the nuclear translocation of STAT1.^48^
ORF7a	Inhibits the IFN-I response by blocking STAT2.	Apoptosis	(+)	Recruits Bcl-XL to the ER, which activates cellular ER stress.^46^
		Pyroptosis	(+)	Promotes the activation of the NF-κB pathway by interacting with TAK1 and IKK complexes.^99,100^
ORF7b	Phosphorylates STAT1 and IRF3.	Apoptosis	(+)	Promotes the phosphorylation of IRF3 and STAT1, which activates TNF-α secretion.^47^
NSP1	Suppresses host gene expression by ribosome association.	Pyroptosis	(−)	Inhibits the activity of caspase-1.^96^
NSP6	Generates the replication organelles.	Pyroptosis	(+)	Promotes NF-κB activation through interactions with TAK1 and IKK complexes.^99,100^
NSP12	RNA-dependent RNA polymerase.	Necroptosis	(+)	Interacts with and promoting RIPK1 activation.^77^
NSP13	RNA helicase	Pyroptosis	(−)	Prevents the formation of GSDMD from caspase-1 cleavage.^96^

*BOK* Bcl-2 ovarian killer, *PDK1* pyruvate dehydrogenase kinase 1, *PKB* protein kinase B, *AKT* serine/threonine kinase, *RNP* ribonucleoprotein, *NLRP3* PYD domain-containing protein 3, *ASC* apoptosis-associated speck-like protein containing a CARD, *NF-κB* nuclear factor kappa B, *TAK1* TGF-beta activated kinase 1, IKK I-kappa-b-kinase, *Bcl-2* B-cell lymphoma-2, *Bax* BCL-2-associated x, *TLR2* toll-like receptor, *IRF3* interferon regulatory factor 3, *IFN* interferon, *STAT1* signal transducer and activator of transcription 1, *ER* endoplasmic reticulum, *TNF-α* tumor necrosis factor-α, *TOM70* translocase of the outer membrane 70, *RIG-I* RNA sensor-I, *MAVS* mitochondrial antiviral signaling, *GSDMD* gasdermin-D, *RIPK1* receptor interacting serine/threonine kinase 1

Given that cell death is important for COVID-19 development, many candidate drugs and treatment strategies have been sought out and investigated via clinical trials (Table 2). The research in this field is advancing and progressing at the molecular level to learn more about the mechanisms of candidate drugs as cell death inducers of virus-infected host cells, and to understand how to use these strategic targets for coordinating and obtaining a high potential treatment and prevention for the rescue of COVID-19 patients. Consequently, knowledge of the mechanisms of various proteins of SARS-CoV-2 in affecting cell death at the molecular level may help identify therapeutic targets against COVID-19.

**Table 2:** Therapeutic strategies associated with cell death of SARS-CoV-2

Drug	Host target	Type of cell death	Mechanism
Infliximab	TNF-α	Apoptosis	Acts as a chimeric IgG1 monoclonal antibody that targets TNF-α.^64,65^
Emricasan	Pan-caspase	Apoptosis	Suppresses caspase-3/7/9.^66,67^
Q-VD	Pan-caspase	Apoptosis	Suppresses caspase-3.^68^
Primidone	RIPK1	Necroptosis	Reduces RIPK1-mediated necroptosis.^83^
Necrostatin-1	RIPK1	Necroptosis	Acts as a RIPK1-targeted inhibitor of necroptosis.^84,85^
MCC950	NLRP3	Pyroptosis	Inhibits SARS-CoV-2 N-induced cytokine production.^97^
Dapansutrile	NLRP3	Pyroptosis	Inhibits the production of early cytokines.^104^
Disulfiram	GSDMD	Pyroptosis	Modifies Cys191/Cys192 in GSDMD to block pore formation, which prevents IL-1β release.^105^
		NETosis	Downregulation of innate immune and complement/coagulation pathways to reduce NET formation.^163,164^
Telmisartan	Angiotensin receptors	Pyroptosis	Inhibits Ang binding with its receptors, which leads to a reduction in the Ang-ATR signaling.^106^
Anakinra	IL-1 receptor	Pyroptosis	Blocks NLRP3 inflammasome-dependent pyroptosis.^107^
Canakinumab	IL-1β	Pyroptosis	A human monoclonal antibody that targets IL-1β.^108^
Tocilizumab	IL-6 receptor	Pyroptosis	An IL-6 receptor antagonist.^110^
	Iron ion	Ferroptosis	Iron chelation.^129^
Imatinib	Free iron	Ferroptosis	Binds with free iron to inactivate iron-containing enzymes and inhibits Fenton reactions.^127^
Selenium	GPX4	Ferroptosis	Preserves GPX4 from irreversible inactivation due to its role in selenocysteine synthesis.^130–133^
Ebselen	GPX4	Ferroptosis	Prevents ferroptosis by acting as a GPX mimic.^132^
	M of SARS-CoV-2		Inhibits viral replication by interacting with SARS-CoV-2 M protein.^131^
N-acetylcysteine	GSH	Ferroptosis	Reinforces the GPX4-GSH-cysteine axis.^136,137^
Ferrostatin-1	Free iron	Ferroptosis	Captures free radicals and slows down the accumulation of PLOOH in PUFA.^138,139^
Liproxstatin-1	Free iron	Ferroptosis	Captures free radicals and slows down the accumulation of PLOOH in PUFA.^138,139^
Sivelestat	NE	NETosis	A selective NE inhibitor.^158^
Cl-amidine	PAD4	NETosis	Reduces histone citrullination in both SARS-CoV-2-infected neutrophils.^149^
Apremilast	PDE4	NETosis	A PDE4 selective inhibitor.^161^
DNase-I	NETs	NETosis	Dissolves thrombogenic NETs.^162^
Dipyridamole	PDE	NETosis	A PDE inhibitor.^171^
Alvelestat	NE	NETosis	Inhibits neutrophil elastase.^173^

*TNF-α* tumor necrosis factor-alpha, *IgG1* immunoglobin G1, *RIPK1* receptor interacting serine/threonine kinase 1, *NLRP3* PYD domain-containing protein 3, *GSDMD* gasdermin-D, *NET* neutrophil extracellular traps, *Ang II* angiotensin II, *ATR* serine/threonine kinase, *IL‑1* interleukin 1, *IL-6* interleukin 6, GPX4 glutathione peroxidase, *GSH* glutathione, *ACSL4* acyl-CoA synthetase long-chain family member 4, *PLOOH* phospholipid hydroperoxides, *PUFA* polyunsaturated fatty acids, *NE* neutrophil elastase, *PAD4* protein arginine deiminase 4, *PDE4* phosphodiesterase 4, *NET* extracellular traps
